# Iron status and anaemia in Sri Lankan secondary school children: A cross-sectional survey

**DOI:** 10.1371/journal.pone.0188110

**Published:** 2017-11-20

**Authors:** Angela Allen, Stephen Allen, Rexan Rodrigo, Lakshman Perera, Wei Shao, Chao Li, Duolao Wang, Nancy Olivieri, David J. Weatherall, Anuja Premawardhena

**Affiliations:** 1 MRC Weatherall Institute of Molecular Medicine, University of Oxford, Oxford, United Kingdom; 2 Department of Clinical Sciences, Liverpool School of Tropical Medicine, Liverpool, United Kingdom; 3 Faculty of Medicine, University of Kelaniya, Ragama, Sri Lanka; 4 Second Military Medical University, Shanghai, China; 5 Department of Medicine and Public Health Sciences, University of Toronto, Toronto, Canada; Centers for Disease Control and Prevention, UNITED STATES

## Abstract

**Background:**

Iron deficiency, the most common micronutrient disorder and cause of anaemia globally, impairs growth, cognition, behaviour and resistance to infection.

**Methods/Results:**

As part of a national survey of inherited haemoglobin variants in 7526 students from 72 secondary schools purposefully selected from the 25 districts of Sri Lanka, we studied 5912 students with a normal haemoglobin genotype. Median age was 16.0 (IQR 15.0–17.0) years and 3189 (53.9%) students were males. Most students were Sinhalese (65.7%), with fewer Tamils (23.1%) and Muslims (11.2%). Anaemia occurred in 470 students and was more common in females (11.1%) than males (5.6%). Haemoglobin, serum ferritin, transferrin receptor and iron were determined in 1196 students with low red cell indices and a structured sample of those with normal red cell indices (n = 513). The findings were weighted to estimate the frequencies of iron deficiency and iron deficiency anaemia classified according to WHO criteria. Iron depletion (serum ferritin <15ug/ml) occurred in 19.2% and cellular iron deficiency (low serum ferritin and transferrin receptor >28.1 nmol/l) in 11.6% students. Iron deficiency anaemia (cellular iron deficiency with low haemoglobin) occurred in only 130/2794 (4.6%) females and 28/2789 (1.0%) males. Iron biomarkers were normal in 83/470 (14.6%) students with anaemia. In multiple regression analysis, the odds for iron depletion and cellular iron deficiency were about one-third in males compared with females, and the odds for iron deficiency anaemia were about one fifth in males compared to females. Tamil ethnicity and age <16 years increased the risk of all three stages of iron deficiency and living at high altitude significantly reduced the risk of iron depletion.

**Conclusions:**

Low iron status and anaemia remain common problems in Sri Lankan secondary school students especially females, younger students and the socioeconomically disadvantaged Tamil population. More research is needed to identify factors other than low iron status that contribute to anaemia in adolescents.

## Background

Iron deficiency is the most common micronutrient disorder and cause of anaemia. It occurs most frequently in children under five years, females of childbearing age and pregnant women. Low iron stores, even in the absence of anaemia, can impair growth [1,2], cognitive ability [1,2], behaviour [1,2], immune function [1,3,4], resistance to infection [1,4], gastrointestinal function [1,4] and hormone balance [1,5]. The associated general fatigue in children and adults impairs school performance and work capacity respectively [6,7]. During pregnancy, iron deficiency anaemia increases the perinatal risks to mother and baby, and is associated with increased infant mortality [1,8].

Iron staining of bone marrow aspirates, the gold standard measure of iron stores, is invasive, traumatic and not appropriate for population surveys. Therefore, WHO recommends the combination of serum ferritin and serum transferrin receptor levels to classify iron deficiency according to three progressive stages [1,9]:

- *Iron depletion*: low iron stores but physiological functions not impaired: low serum ferritin
- *Cellular iron deficiency*: More marked iron insufficiency, as iron stores are exhausted and normal cellular physiological functions are impaired: low serum ferritin and raised transferrin receptor
- *Iron deficiency anaemia*: persistence of iron deficiency long enough to reduce red cell mass, reflected in reduced haemoglobin (Hb) concentration, low serum ferritin and raised transferrin receptor

As part of a national survey of secondary school students to determine the frequency of Hb variants in Sri Lanka, we determined iron status classified according to these WHO criteria. We also measured serum iron, as a low cost assay in resource-limited settings. We evaluated factors known to be associated with iron status including age [10], sex [11], altitude [12] and ethnicity [13].

## Methods

### School surveys

School surveys took place between June 2009 and July 2010 in the 25 districts of Sri Lanka. From the total of 10,144 schools, we aimed to recruit 300 secondary school students from grades 6 to 13 (equivalent to ages 11–19 years) in each district. Two-three schools were purposefully selected from each district so that they were geographically spaced and their students represented the major ethnic groups. We included 5 temporary secondary schools established in camp settlements in the north of the island for children displaced from the two neighbouring districts of Mullathivu and Kilinochichi following the end of the civil war in May 2009. We selected a total of 72 secondary schools.

Each school was visited prior to study enrolment, to explain the purpose of the study to parents, students and teachers. All students were invited to enrol for the study and were allowed to decline if they did not wish to participate. Written, signed consent was obtained from parents/guardians for all students, and students also provided verbal consent. Students were recruited on a “first come, first served” basis drawn from across the seven school years, until approximately 300 students from each district had been enrolled. Each student’s gender, date of birth, residence and ethnic group as reported by the student was recorded. In those with mixed ethnicity, that of the father was recorded. Any student who felt unwell on the day of the survey was not enrolled in the study.

### Laboratory procedures

5ml venous blood was collected from each student; 2.5 ml was transferred into an EDTA anticoagulated tube, and the remaining sample to a plain tube. The EDTA sample was used for the detection of haemoglobin variants using the β-thalassaemia short course program on the BioRad Haemoglobin variant analyzer 1 (Bio-Rad, Chennai, India). Haemoglobin and red cell indices were measured using a haematology analyser (Beckman Coulter Ac.T diff analyzer, Luton, UK). The remaining EDTA sample was centrifuged and the plasma separated from the cell pellet.

The blood sample transferred to the plain tube was allowed to clot, centrifuged and the serum separated and transferred into two 1.5ml screw capped Eppendorf vials.

Cell pellets, plasma and sera samples were then stored at -20°C until further analysis.

Based on recommended guidelines [14–16], students were classified as having low red cell indices if they had a mean cell volume (MCV) <80fl and/or a mean cell haemoglobin (MCH)<27 pg. In accordance with WHO guidelines anaemia was defined as Hb <11.5.0 g/dl in children < 12 years, Hb <12.0 g/dl in females ≥12 years and males aged 12–14 years and Hb <13.0 g/dl in males aged 15 years and above [17]. Haemoglobin values were also adjusted by -0.5 g/dl for students from Nuwara Eliya which is 1868m above sea level (17). Biomarkers of iron status were determined in all students with low red cell indices to guide treatment for iron deficiency on an individual basis. To investigate iron status in students with normal red cell indices and limited by available funding, we selected every 10^th^ consecutive sample for analysis. This resulted in approximately 20 samples (likely 10 girls and 10 boys) from each of the 25 districts and 513 samples in total.

Serum was shipped to UK on dry-ice. Serum iron was measured using a manual colorimetric assay (SI 257, Randox laboratories, County Antrim, UK), serum ferritin by ELISA immunoassay (DB59111, IBL International, Hamburg, Germany) and serum transferrin receptor using ELISA immunoassay (DTFR1, R and D Systems, Abingdon, UK). All assays were run in duplicate. Cut-off values used to define iron deficiency were: ferritin <15ng/ml, in accordance with WHO recommendations [17] and for transferrin receptor >28.1nmol/l and serum iron <10.6μmol/l in males and <6.6 μmol/l in females, in accordance with the kit manufacturers’ guidelines.

DNA was extracted from the EDTA cell pellet using either QIAGEN DNA mini kit or phenol chloroform extraction, and alpha globin genotype was determined by GAP PCR [18].

### Statistical analysis

Students with a haemoglobinopathy trait, missing gender or haemoglobin value or insufficient sample for further analysis were excluded. Hb and red cell indices were reported for all other students using medians, interquartile range and analysed according to demographic variables using non-parametric tests.

In view of the incomplete testing of all students with normal red cell indices for iron biomarkers, a weighted analysis was used to estimate the frequency of iron deficiency and iron deficiency anaemia in the whole student sample. Based on the sample distributions and number of blood samples available, weights were 9.170 for the group with normal red cell indices and 1.004 for the low red cell indices group. These weights were applied when calculating the summary statistics and performing logistic regression analysis to identify demographic variables independently associated with iron status and iron-deficiency anaemia. Age was separated into two groups of approximately equal numbers: <16 years and 16 years and above, and to likely differentiate between the pre and post pubertal growth phase in all children and pre and post-menarchy in girls. Altitude ranged from 0-1868m above sea level and was grouped as <500m, 500-1000m and >1000m. All data analysis was performed using SPSS and STATA statistical software, versions 23 and 14, respectively.

## Ethical approval

The study and the consent procedures were approved by the Ethical Committee, University of Kelaniya, Sri Lanka and Oxford University Tropical Research Committee, Oxford, UK.

## Results

**Fig 1:**
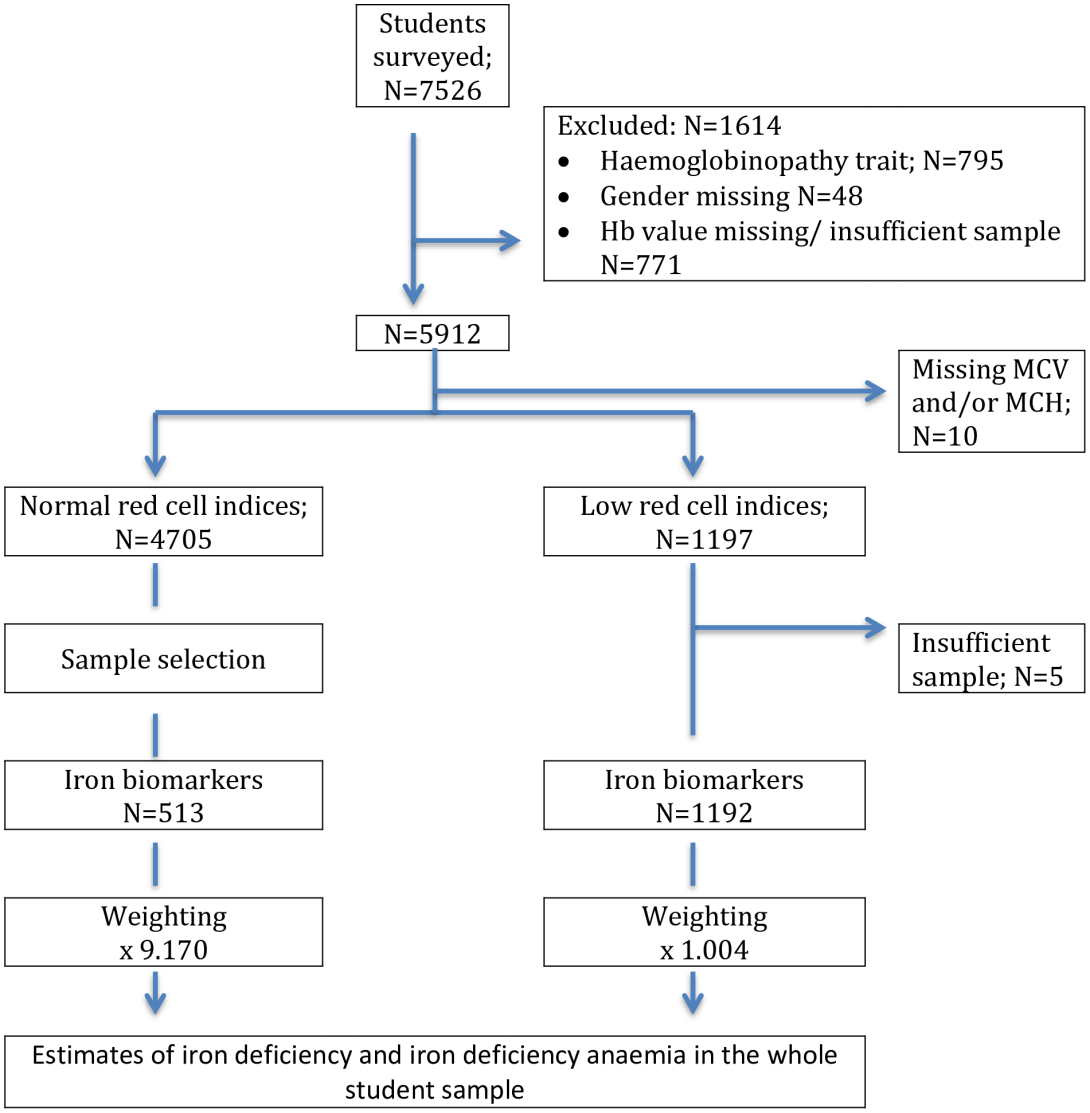
Flow of participants through the study.

A total of 7526 students were enrolled in the study. In addition to students with a haemoglobin variant, we also excluded students with missing gender or a haemoglobin value or insufficient sample for further analysis (Fig 1). Haemoglobin and red cell indices were measured in 5912 of whom 3189 (53.9%) were males (Table 1). The median age of the students was 16.0 (IQR 15.0–17.0) years; age was missing in 140 students. 3884 (65.7%) students were Sinhalese ethnicity, 1367 (23.1%) Tamil and 660 (11.2%) Muslim; ethnicity data was missing for 1 student.

**Table 1:** Haemoglobin concentration and red cell indices according to demographic variables.

Covariates	Hb g/dl		MCV fl		MCH pg		RDW fl		RBC x10^12^/L	
	N Median (IQR)	P value	N Median (IQR)	P value	N Median (IQR)	P value	N Median (IQR)	P value	N Median (IQR)	P value
**Gender**										
Male	3189 15.03 (14.00–16.00)	<0.001	3186 84.70 (82.40–87.30)	0.179	3188 29.10 (27.80–30.70)	0.047	3183 13.10 (12.70–13.60)	0.009	3188 5.14 (4.88–5.40)	0.001
Female	2723 13.34 (12.60–14.20)		2716 84.75 (81.80–87.60)		2721 29.40 (27.80–31.00)		2715 13.10 (12.60–14.00)		2721 4.59 (4.38–4.81)	
**Age**										
≥16	3730 14.52 (13.40–15.60)	<0.001	3722 85.10 (82.60–87.90)	<0.001	3729 29.80 (28.30–31.30)	<0.001	3717 13.10 (12.60–13.70)	<0.001	3729 4.89 (4.54–5.25)	<0.001
<16	2042 13.81 (12.90–14.80)		2041 84.10 (81.50–86.60)		2042 28.40 (27.30–29.90)		2042 13.20 (12.70–13.90)		2041 4.82 (4.57–5.12)	
**Ethnicity**										
Sinhalese	3884 14.44 (13.30–15.60)	<0.001	3875 84.60 (82.10–87.40)	0.007	3883 29.70 (28.10–31.30)	<0.001	3875 13.10 (12.60–13.80)	0.004	3883 4.86 (4.53–5.22)	<0.001
Muslim	660 13.97 (12.90–14.90)		659 84.60 (82.10–87.40)		660 28.80 (27.60–30.20)		659 13.10 (12.70–13.70)		660 4.82 (4.54–5.13)	
Tamil	1367 13.89 (12.90–14.90)		1367 85.10 (82.50–87.70)		1367 28.40 (27.30–29.50)		1363 13.20 (12.70–13.80)		1366 4.91 (4.65–5.19)	
**Altitude** (m)										
<500	4941 14.17 (13.10–15.20)	<0.001	4931 84.60 (82.00–87.20)	<0.001	4940 29.20 (27.70–30.90)	0.008	4927 13.20 (12.70–13.80)	<0.001	4939 4.83 (4.53–5.16)	<0.001
500–1000	764 14.86 (13.90–15.90)		764 85.60 (83.00–88.48)		764 29.40 (28.10–30.80)		764 13.00 (12.50–13.50)		764 5.05 (4.75–5.38)	
>1000	207 14.08 (13.40–15.00)		207 87.00 (84.00–90.40)		207 29.50 (28.40–30.60)		207 12.90 (12.40–13.60)		207 5.00 (4.71–5.25)	
**TOTAL**	5912 14.26 (13.20–15.30)		5902 84.70 (82.20–87.50)		5909 29.20 (27.80–30.90)		5898 13.10 (12.70–13.80)		5909 4.87 (4.56–5.20)	

Hb = haemoglobin; MCV = mean cell volume; MCH = mean cell haemoglobin concentration; RDW = red cell distribution width; RBC = red blood cell count

**Fig 2:**
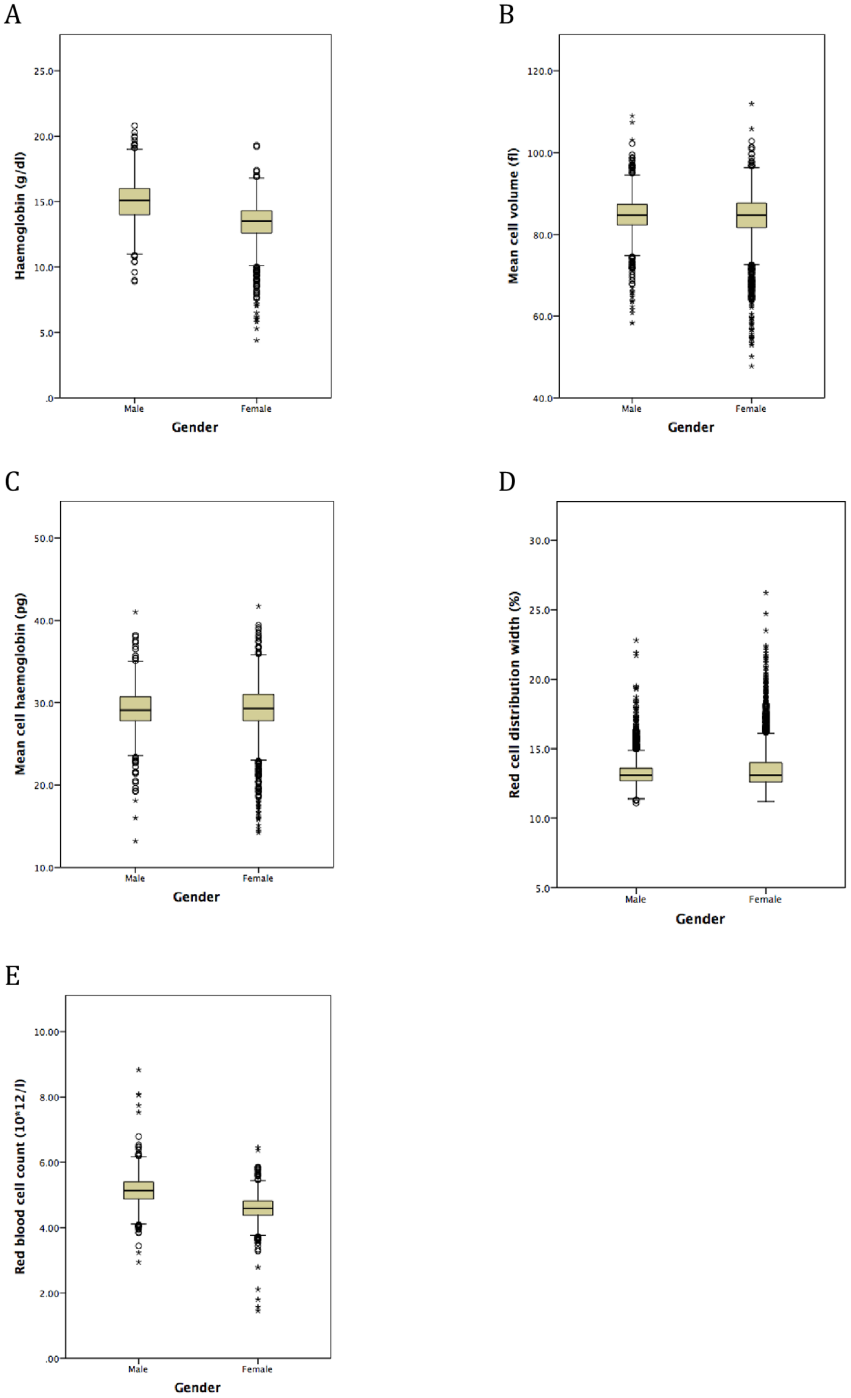
Box plots to show haemoglobin and red cell indices according to gender in secondary school students from Sri Lanka: (A) haemoglobin; (B) mean cell volume; (C) mean cell haemoglobin; (D) red cell distribution width; (E) red blood cell count. Horizontal lines inside the box show the median value, box length is the interquartile range and whiskers show the range, excluding outliers. Outlying values 1.5 to 3, or > 3 box lengths from the upper and lower edge of the box are shown as open circles and stars respectively.

Median Hb was lower in females (13.3 g/dl) than males (15.0 g/dl; p<0.001, Table 1, Fig 2a). Overall, anaemia occurred in 470 (8.1%) students and was more common in females (11.1%) than males (5.6%, S1 Table). One male (0.031%) and fifteen females (0.54%) were severely anaemic (Hb <9.0 g/dl in males and Hb <8.0 g/dl in females).

Serum ferritin, transferrin receptor and iron were measured in 1196 students with low red cell indices and in 513 with normal red cell indices and these results were weighted to estimate the frequencies of iron deficiency and iron deficiency anaemia for the whole student population sample (Table 2). Table 3 shows the progressive stages of iron deficiency using WHO criteria according to demographic variables in this weighted population. Overall, iron depletion (low serum ferritin) was present in 1105 (19.2%) students, cellular iron deficiency (low serum ferritin and raised serum transferrin receptor) in 662 (11.6%) and iron deficiency anaemia (cellular iron deficiency with low haemoglobin according to gender) in 217 (3.9%) students.

**Table 2:** Iron biomarkers according to demographic variables.

Variable	Serum Ferritin ng/ml	P value	Serum transferrin receptor nmol/l	P value	Serum Iron μmol/l	P value
	N Median (IQR)		N Median (IQR)		N Median (IQR)	
**Gender**						
Boys	2876 41.69 (25.25–59.83)	<0.001	2880 25.74 (21.90–30.50)	<0.001	2700 17.61 (14.01–21.19)	<0.001
Girls	2885 26.63 (13.82–38.88)		2914 25.02 (20.12–30.67)		2830 14.14 (10-.28–18.13)	
**Age**						
≥16	3343 33.44 (19.38–54.10)	<0.001	3367 24.72 (20.30–29.93)	<0.001	3206 16.38 (12.36–20.51)	<0.001
<16	2304 31.07 (18.59–45.95)		2303 26.66 (21.68–31.99)		2209 15.71 (11.67–19.20)	
**Ethnicity**						
Sinhalese	3697 33.04 (19.52–51.40)	<0.001	3723 25.41 (20.88–30.67)	<0.001	3589 16.90 (12.67–20.69)	<0.001
Muslim	595 35.35 (18.51–57.26)		602 23.67 (19.82–27.48)		555 16.50 (11.64–19.19)	
Tamil	1469 29.12 (17.73–45.25)		1468 26.04 (21.32–32.26)		1387 14.91 (11.18–18.50)	
**Altitude (m)**						
<500	4818 32.87 (19.52–51.29)	0.003	4840 25.43 (20.73–30.99)	0.311	4652 16.17 (12.25–19.76)	0.003
500–1000	693 31.29 (16.58–46.87)		703 25.07 (21.14–29.55)		648 17.09 (10.80–21.14)	
>1000	250 33.21 (25.15–51.68)		250 26.77 (22.98–30.67)		230 16.07 (10.57–19.22)	
**Overall**	**5761** **32.68 (19.35–51.29)**		**5794** **25.30 (20.91–30.58)**		**5530** **16.25 (12.19–19.97)**	

**Table 3:** Progressive stages of iron deficiency according to demographic variables.

Covariates	Iron depletion			Cellular iron deficiency			Iron deficiency anaemia		
	N (%)	OR (95% CI)	P value	N (%)	OR (95% CI)	P value	N (%)	OR (95% CI)	P value
**Gender**		-	-		-	-		-	-
Female	783/2885 (27.1)	ref	-	495/2843 (17.4)	ref	-	130/2794 (4.6)	ref	-
Male	322/2876 (11.2)	0.32 (0.28–0.37)	<0.001*	167/2857 (5.8)	0.28 (0.23–0.33)	<0.001*	28/2785 (1.0)	0.20 (0.13–0.31)	<0.001*
**Age**		-	0.013		-	<0.001*		-	0.008*
≥16	627/3343 (18.8%)	ref	-	323/3303 (9.8)	ref	-	78/3297 (2.4)	ref	-
<16	475/2304 (20.6%)	1.19 (1.04–1.37)		336/2281 (14.7)	1.74 (1.47–2.06)		79/2281 (3.5)	1.55 (1.12–2.14)	
**Ethnicity**		-	0.002*		-	<0.001*		-	0.002*
Tamil	312/1470 (21.2)	ref	-	214/1454 (14.7)	ref	-	61/1401 (4.3)	ref	-
Sinhalese	690/3697 (18.7)	0.75 (0.64–0.88)	<0.001	420/3650 (11.5)	0.73 (0.61–0.88)	0.001	89/3592 (2.5)	0.57 (0.40–0.80)	0.001
Muslim	104/595 (17.5)	0.81 (0.63–1.04)	0.104	27/595 (4.5)	0.28 (0.19–0.43)	<0.001	8/586 (1.4)	0.33 (0.16–0.70)	0.003
**Altitude (m)**		-	<0.001*		-	0.703*		-	0.674
>1000	33/250 (13.2)	ref	-	31/250 (12.4)	ref	-	8/250 (3.2)	ref	-
500–1000	152/693 (21.9)	2.64 (1.73–4.03)	<0.001	74/693 (10.7)	1.21 (0.76–1.92)	0.431	11/625 (1.8)	0.84 (0.33–2.18)	0.726
<500	920/4818 (19.1)	1.65 (1.13–2.42)	0.010	557/4756 (11.7)	1.10 (0.74–1.64)	0.639	139/4703 (3.0)	1.12 (0.54–2.34)	0.763
**Total**	1105/5761 (19.2)	-	-	662/5699 (11.6)	-	-	217/5368 (3.9)	-	-

* P value for the variable in multiple regression analysis

The strongest risk factor for iron deficiency was gender, with the odds for all three stages of iron deficiency being three or more times likely in females than males (Table 3). All 3 stages of iron deficiency were more common amongst younger than older students and were most common amongst Tamils and least common in Muslims. Living at high altitude significantly reduced the odds of iron depletion but was not statistically significantly associated with cellular iron deficiency or iron deficiency anaemia. In multiple regression analysis, gender, age and ethnicity were independently associated with all three stages of iron deficiency and altitude was independently associated with iron depletion (Table 3).

As expected, low red cell indices were generally associated with iron deficiency. However, median MCV and MCH values were similar in both genders (Table 1, Fig 2b and 2c). The median RDW value was the same for both genders but there was a preponderance of females amongst students with higher values (Table 1, Fig 2d). Very few students had MCV values above the normal range (>96 fl; Fig 2b). Also, Tamils had the highest median MCV value, despite the greatest frequency of all three stages of iron deficiency (Table 1).

Interestingly, median serum transferrin receptor was lower (Table 2) in females than males despite their markedly worse iron status.

Median serum ferritin levels were significantly higher but, conversely, iron levels significantly lower in students residing at high altitude (>1000m). Serum transferrin receptor levels were also higher at high altitude, although this was not statistically significant.

Marked differences in the frequency of anaemia, low red cell indices and the stages of iron deficiency were observed between regions of Sri Lanka (S1 and S2 Tables). Anaemia and iron deficiency were greater in the northern regions of the island.

Anaemia as measured in the whole population sample (defined by low Hb according to gender; 8.1%) was markedly more common than the estimate of iron deficiency anaemia (defined by low Hb, low ferritin and raised serum transferrin receptor; 3.9%) based on the weighted population. Of those students with anaemia in the weighted population, serum ferritin was normal but transferrin receptor was raised in 29.9% males and 12.4% females. However, in these anaemic students, serum ferritin and transferrin receptor levels were normal in 23.4% males and 12.0% females and all three iron biomarkers were normal in 16.4% males and 9.1% females.

## Discussion

As far as we are aware, this is the most comprehensive study of iron status and iron deficiency anaemia amongst adolescent students in Sri Lanka reported to date. In otherwise healthy adolescents and without haemoglobinapthy traits, iron depletion, identified by low serum ferritin, affected more than 1 in 4 females and 1 in 10 males. Iron deficiency was sufficiently severe to cause cellular iron deficiency, indicated by a raised serum transferrin receptor as well as low ferritin, in about 1 in 6 females and 1 in 17 males. Using the WHO classification, anaemia could be ascribed to iron deficiency in about 1 in 22 females and 1 in 100 males. Persistence of low iron status is despite significant urbanisation and strong economic growth in Sri Lanka in recent years, which is now classified as a lower middle-income country [19]. In addition, this study was performed when malaria control had reduced transmission to very low levels ahead of malaria eradication in 2016 [20].

Female gender was the greatest risk factor for low iron status; each of the three stages of iron deficiency was at least three times more common in females than males. Although there are few studies of iron status in Sri Lankan adolescents, our findings are consistent with a study in the Western Province where low serum ferritin was found in 29.4% adolescent female school drop-outs [21]. The age at which girls achieve menarche varies worldwide [22] and in Sri Lanka occurs between the ages of 11–13 years [23]. The greater frequency of iron deficiency and iron deficiency anaemia in females compared with males is likely due to insufficient iron intake to compensate for menstrual losses [24–27].

Although Hb levels were lower in females, median MCV was similar in both sexes. Although very few of our students had macrocytosis, deficiencies of folate and/or vitamin B_12_ may have raised red cell indices in students with iron deficiency. We were unable to measure these haematinics in our study due to limited sample volume, but previous studies in Sri Lanka found 45.1% of anaemic adolescent females in Colombo [28] and 28% adolescent female school dropouts in Kalutara and Colombo, Western province were folate deficient [21]. A study from Galle district, Southern region, showed that the frequency of folate deficiency was 41% in males and 33% in females [29]. Differences in folate or vitamin B_12_ status may also have accounted for differences in MCV observed according to ethnicity.

All stages of iron deficiency were more common in younger students. This may be due to the increased demand for iron during the most active phase of the pubertal growth spurt that occurs before age 16 years in most children [30,31]. In Sri Lanka most adolescent girls have completed puberty by age 15.5 years [23].

Lower Hb levels and poorer iron status were observed in districts in the north of the island. We also observed a high frequency of iron deficiency in the southern province, which is in keeping with a previous study of 945 adolescents from Galle district where 49.5% of males and 58.1% females were anaemic (Hb <12.0g/dl), and of those who were anaemic 30.2% males and 47.8% females had low iron stores (ferritin <30 ng/ml) [29]. We observed less anaemia and better iron status in the Galle population than in the previous study. This may be due to differences in laboratory methods and choice of cut-off values for ferritin, or an improvement in iron status over time.

Iron deficiency and iron deficiency anaemia were more common in Tamils than the other ethnic groups. To our knowledge, constitutional differences in Hb according to ethnicity have not been reported previously in Sri Lanka but have been observed in other populations. In the United States, Hb levels were typically 5-10g/l lower in blacks than whites [32–34] and this was independent of iron status [34]. Ethnic differences in serum transferrin receptor have been reported from the United States in a study of 225 healthy, haematologically normal adults, where levels were about 9% higher in blacks than Caucasians [35]. Alternatively, worse iron status amongst Tamils is also likely to reflect the socio-economic disadvantage of this population. The Tamil students were previously most affected by war and were residing in temporary camps where resources were limited and malnutrition was common [36–38]. Due to the marked geographical differences in ethnic composition of the population across the island, we were not able to distinguish ethnicity from district or region of residence as risk factors for iron deficiency and anaemia. Tamil students were mainly from the northern regions where poverty persists (as well as in the East, Estate Sector and Moneragala district) and there are less opportunities of access to services and the labour market. (http://www.worldbank.org/en/country/srilanka/overview) [19].

The associations between altitude and Hb, red cell indices and biomarkers of iron status are difficult to interpret. The higher ferritin levels at higher altitude suggesting greater iron stores contrast with the higher serum transferrin receptor levels suggesting greater cellular iron deficiency. The frequency of iron deficiency anaemia did not differ significantly with altitude (p = 0.71). Although serum ferritin levels are not directly influenced by altitude [12], there will be an additional iron requirement due to the increased erythropoiesis, driven by the relative hypoxia, to ensure a sufficient supply of oxygen to the tissues [39], and this may explain the higher transferrin receptor levels irrespective of iron status. Allen et al found that in healthy, haematologically normal adults, in the USA, serum transferrin receptor levels were about 9% higher in those living at 1600m above sea level, than in those residing at altitudes below 300m [35].

Studies in non-malarious areas have reported differences in iron status and anaemia according to season. Jiang et al showed that pregnant women in Nepal were most iron deficient during the monsoon season [40]. In Sri Lanka, monsoons tend to occur between May and September in the South-West region, and between October and January in the North and eastern coastal regions of the country. Since it was not possible to ensure that students from each district were sampled equally during every season we were not able to evaluate the effects of season on iron status and anaemia.

A notable finding in our study was that despite excluding all students with haemoglobinopathy traits from the analysis, low haemoglobin values were still more common than iron deficiency anaemia, classified according to the WHO criteria.

As discussed previously, deficiencies in folate and/or vitamin B_12_ may also account for the markedly higher frequency of anaemia than iron deficiency anaemia in both boys and girls. Although a range of laboratory tests are available for iron deficiency and iron deficiency anaemia, each test has its limitations and the relationships between the biomarkers are complex. A well-known difficulty is the assessment of iron status in settings where infection is common [41]. Serum ferritin is an acute phase protein and would be elevated as a consequence of infection, potentially masking low iron stores [17,41]. Although our finding that serum transferrin receptor was raised in many students with normal ferritin values is consistent with this, our study population were healthy adolescents attending school, and in whom acute infections were likely to be uncommon. Indeed, blood samples were not collected from any child who felt unwell on the day of the survey. Furthermore, measurement of C-reactive protein (CRP) in 2263 students from the survey revealed that only 3.5% had a value greater than the upper range of normal (5 mg/l); confirming that inflammation was uncommon [42]. Rather than inflammation, starvation or fasting for even a short period can also cause an increase in the serum ferritin concentration [17]. We did not record time since last meal in our study and so cannot exclude this possibility.

It is of interest that we estimated that all 3 biomarkers of iron status were normal in 16.4% males and 9.1% females with anaemia. We plan to further investigate correlations between Hb, red cell indices and iron biomarkers, including values used for reference ranges, at the individual level to further explore possible causes of low haemoglobin values.

The complexity in interpreting different iron indices at the population level is well illustrated by our study. Although several of the differences in red cell indices and iron biomarkers according to demographic variables that we observed were small and unlikely to be of clinical significance, they were highly statistically significant in our large population sample. Furthermore, these small differences are sensitive to the specific cut-offs that are used to determine prevalence of iron deficiency and anaemia at the population level.

According to the WHO recommendation, our population of students should be classified as “iron deficiency is prevalent; inflammation is prevalent” based on the prevalence of low serum ferritin <20% and high serum transferrin receptor >10% [9,17]. Although serum transferrin receptor may overestimate iron deficiency in populations in whom inflammation is common, for example in malaria endemic regions [43], as reported above, we do not consider that inflammation was prevalent in our study. Interestingly, serum transferrin receptor levels in our study were statistically significantly higher in males than females despite the markedly worse iron status of females. Similar findings were reported in Spanish adolescents [44]. The manufacturers of the assay kit recommend adjusting the normal range according to ethnicity (https://resources.rndsystems.com/pdfs/datasheets/dtfr1.pdf), and reference ranges for serum transferrin receptor have not been established in the Sri Lankan population. More research is needed to determine reference values for serum transferrin receptor according to gender and in different populations.

Our study had several weaknesses. A significant number of students were excluded because of missing Hb values; however, this was due to instrument failure or insufficient sample volume rather than any bias in student selection. Although we did not collect data on students who declined to volunteer to participate, students were keen to know their status regarding inherited haemoglobinopathies and, therefore, we believe that refusals to participate in the study were uncommon. We were able to measure iron biomarkers in only approximately 10% of students with normal red cell indices. As a result, we performed a weighted analysis to estimate iron deficiency and iron-deficiency anaemia in the whole student sample. However, we selected a systematic sample of these students to minimise possible selection bias.

In conclusion, both iron deficiency and anaemia were common in this healthy adolescent population in Sri Lanka. In addition to gender, a well-known risk factor for iron deficiency, we found that age <16 years and Tamil ethnicity increased the risk of all three stages of iron deficiency and living at high altitude significantly reduced the risk of iron depletion. Iron deficiency could not account for anaemia in 16.4% males and 9.1% females; causes of anaemia other than iron deficiency require further investigation in this population. Our study also highlights that reference ranges for serum transferrin receptor in specific populations need to validated to support its use alongside serum ferritin in assessing the prevalence and severity of iron deficiency at the population level. These findings in Sri Lanka may also be relevant to similar adolescent populations in South Asia but further research should be undertaken to identify risk factors relevant to specific populations.

## Supporting information

S1 File Haemoglobin and red cell indices database. (XLSX)

S2 File Iron biomarker database. (XLSX)

S1 Table Low red cell indices and anaemia in males and females according to district. (DOCX)

S2 Table Progressive stages of iron deficiency in males and females according to province and district. (DOCX)
