# Supplementary material for: Iron status and anaemia in Sri Lankan secondary school children: A cross-sectional survey
Source: PLoS One. 2017 Nov 20;12(11):e0188110. doi: 10.1371/journal.pone.0188110 (PMC5695819; doi:10.1371/journal.pone.0188110)
Supplement: S1 Table — (DOCX) [file pone.0188110.s003.docx]

**Table S1: Low red cell indices and anaemia in males and females according to district**

| **Province** | **District** | **Low red cell indices**  (MCV< 80 fl and /or MCH<27 pg) | | **Anaemia**  (Hb<11.5 g/dl in all children <12 years, Hb <12.0 g/dl in males 12-14 years and females ≥ 12 years, and Hb<13.0 g/dl in males ≥15 years) | |
| --- | --- | --- | --- | --- | --- |
|  |  | **Males**  **N (%)** | **Females**  **N (%)** | **Males**  **N (%)** | **Females**  **N (%)** |
| Northern* | Jaffna | 36/226 (15.9) | 12/56 (21.4) | 38/223 (17.0) | 11/56 (19.6) |
|  | Vavuniya | 47/174 (27.0) | 30/88 (34.1) | 10/174 (5.8) | 22/88 (25.0) |
|  | Mannar | 55/161 (34.2) | 26/96 (27.1) | 26/157 (16.6) | 15/94 (16.0) |
|  | Kilinochchi | 16/44 (36.4 ) | 21/50 (42.0) | 1/44 (2.3) | 5/49 (10.2) |
| **Total** |  | **154/605 (25.5)** | **89/290 (30.7)** | **75/598 (12.5)** | **53/287 (18.5)** |
| North Central | Anuradhapura | 23/100 (23.0) | 46/141 (32.6) | 7/100 (7.0) | 26/140 (18.6) |
|  | Polonnaruwa | 29/117 (24.8) | 43/157 (27.4) | 6/117 (5.1) | 23/157 (14.7) |
| **Total** |  | **52/217 (24.0)** | **89/298 (29.9)** | **13/217 (6.0)** | **49/298 (16.5)** |
| North West | Puttalam | 26/81 (32.1) | 73/181 (40.3) | 8/81 (9.9) | 43/181 (23.8) |
|  | Kurunegala | 15/92 (16.3) | 24/141 (17.0) | 0/92 (0.0) | 4/141 (2.8) |
| **Total** |  | **41/173 (23.7)** | **97/322 (30.1)** | **8/173 (4.6)** | **47/275 (14.6)** |
| East | Trincomalee | 30/199 (15.1) | 16/75 (21.3) | 18/193 (9.3) | 10/74 (13.5) |
|  | Batticaloa | 20/179 (11.2) | 18/84 (21.4) | 7/176 (4.0) | 7/84 (8.3) |
|  | Ampara | 45/177 (25.4) | 21/82 (25.6) | 12/82 (14.6) | 14/67 (20.9) |
| **Total** |  | **95/555 (17.1)** | **55/241 (22.8)** | **37/451 (8.2)** | **31/225 (13.8)** |
| Central | Matale | 19/153 (12.4) | 28/114 (24.3) | 3/153 (2.0) | 14/114 (12.3) |
|  | Kandy | 21/156 (13.5) | 8/51 (15.7) | 1/156 (0.6) | 3/51 (5.9) |
|  | Nuwara Eliya | 6/117 (5.1) | 16/90 (17.8) | 5/117 (4.3) | 8/90 (8.9) |
| **Total** |  | **46/426 (10.8)** | **52/255 (20.4)** | **9/426(2.1)** | **25/255 (9.8)** |
| West | Gampaha | 9/63 (14.3) | 38/163 (23.3) | 2/63 (3.2) | 17/167 (10.2) |
|  | Colombo | 42/172 (24.4) | 42/117 (35.9) | 11/172 (6.4) | 26/117 (22.2) |
|  | Kalutara | 13/85 (15.3) | 29/187 (15.0) | 3/85 (3.5) | 5/187 (2.7) |
| **Total** |  | **64/320 (20.0)** | **109/467 (23.3)** | **16/320 (5.0)** | **48/471 (10.2)** |
| Sabaragamuwa | Ratnapura | 9/134 (6.7) | 21/144 (14.6) | 2/134 (1.5) | 1/144 (0.7) |
|  | Kegalle | 16/103 (15.5) | 36/151 (23.3) | 4/101 (4.0) | 21/148 (14.2) |
| **Total** |  | **25/237 (10.5)** | **57/295 (19.3)** | **6/235 (2.6)** | **22/292(7.5)** |
| Uva | Badulla | 28/192 (14.5) | 10/98 (10.2) | 4/192 (2.1) | 3/98 (3.1) |
|  | Moneragala | 25/94 (26.6) | 20/93 (21.5) | 1/94 (1.1) | 3/93 (3.2) |
| **Total** |  | **53/286(18.5)** | **30/191 (15.7)** | **5/286 (1.8)** | **6/191 (3.1)** |
| South | Galle | 4/85 (4.7) | 14/120 (11.7) | 1/85 (1.2) | 3/121 (2.4) |
|  | Hambantota | 12/93 (12.9) | 28/156 (17.9) | 1/93 (1.1) | 9/156 (5.8) |
|  | Matara | 23/191 (12.0) | 11/81(12.6) | 1/192 (0.5) | 5/81 (6.2) |
| **Total** |  | **39/369 (10.6)** | **53/357 (14.8)** | **3/370 (0.8)** | **17/358 (4.8)** |
| **Overall total** |  | **569/3188 (17.8)** | **631/2716 (23.2)** | **172/3076 (5.6)** | **298/2698 (11.1)** |

* Haemoglobin and red cell indices data for Mullathievu were incomplete and therefore omitted from the table.
