# Supplementary material for: Iron status and anaemia in Sri Lankan secondary school children: A cross-sectional survey
Source: PLoS One. 2017 Nov 20;12(11):e0188110. doi: 10.1371/journal.pone.0188110 (PMC5695819; doi:10.1371/journal.pone.0188110)
Supplement: S2 Table — (DOCX) [file pone.0188110.s004.docx]

**Table S2: Progressive stages of iron deficiency in males and females according to province and district**

| **Province** | **District** | **Iron depletion** | |  | **Cellular iron deficiency** | |  | **Iron deficiency anaemia** | |
| --- | --- | --- | --- | --- | --- | --- | --- | --- | --- |
|  |  | Males  N (%) | Females  N (%) |  | Males  N (%) | Females  N (%) |  | Males  N (%) | Females  N (%) |
| Northern | Jaffna | 37/147 (25.2) | 40/83 (48.2) |  | 15/146 (10.3) | 31/83 (37.3) |  | 13/146 (9.0) | 12/82 (14.8) |
|  | Vavuniya | 21/157 (13.4) | 46/126 (36.5) |  | 18/157 (11.5) | 27/120 (22.5) |  | 0/157 (0) | 4/120 (3.4) |
|  | Mannar | 17/148 (11.5) | 29/151 (19.2) |  | 16/148 (10.8) | 24/151 (15.9) |  | 5/145 (3.5) | 3/151 (2.0) |
|  | Mullaitivu | 1/38 (2.6) | 8/50 (16.0) |  | 1/38 (2.6) | 8/50 (16.0) |  | 0/28 (0) | 3/34 (8.8) |
|  | Kilinochchi | 25/92 (27.2) | 44/121 (36.4) |  | 18/92 (19.6) | 23/112(20.5) |  | 0/91 (0) | 9/112 (8.1) |
| **Total** |  | **101/582 (17.4)** | **167/531 (31.5)** |  | **68/581 (11.7)** | **113/516 (21.9)** |  | **18/568 (3.2)** | **31/500 (6.3)** |
| North Central | Polonnaruwa | 5/144 (3.5) | 49/167 (29.3) |  | 2/143 (1.4) | 27/166 (16.3) |  | 1/143 (0.7) | 11/166 (6.6) |
|  | Anuradhapura | 34/166 (20.5) | 20/120 (16.7) |  | 23/165 (13.9) | 16/120 (13.3) |  | 2/166 (1.2) | 12/111 (10.9) |
| **Total** |  | **39/310 (12.6)** | **69/287 (24.0)** |  | **25/308 (8.1)** | **43/286 (15.0)** |  | **3/308 (1.0)** | **23/277 (8.3)** |
| North West | Puttalam | 30/146 (20.5) | 66/174 (37.9) |  | 1/145 (0.7) | 33/174 (19.0) |  | 1/145 (0.7) | 11/174 (6.3) |
|  | Kurunegala | 0/112 (0) | 33/85 (38.8) |  | 0/112 (0) | 4/84 (4.8) |  | 0/112 (0) | 2/84 (2.4) |
| **Total** |  | **30/258 (11.6)** | **99/259 (38.2)** |  | **1/257 (0.4)** | **37/258 (14.3)** |  | **1/257 (0.4)** | **13/258 (5.1)** |
| East | Trincomalee | 2/135 (1.5) | 8/133 (6.0%) |  | 2/133 (1.5) | 7/133 (5.3) |  | 0/133 (0) | 5/133 (3.8) |
|  | Batticaloa | 1/126 (0.8) | 5/114 (4.4%) |  | 1/126 (0.8) | 5/114 (4.4) |  | 1/124 (0.8) | 4/113 (3.6) |
|  | Ampara | 21/127 (16.5) | 19/139(13.7%) |  | 3/128 (2.3) | 7/139 (5.0) |  | 0/128 (0) | 6/139 (4.3) |
| **Total** |  | **24/388 (6.2)** | **32/386 (8.3)** |  | **6/387 (1.6)** | **19/386 (4.9)** |  | **1/385 (0.3)** | **15/385 (3.9)** |
| Central | Matale | 19/135 (14.0) | 57/136 (41.9) |  | 1/135 (0.7) | 28/136 (20.6) |  | 0/135 (0) | 6/136 (4.4) |
|  | Kandy | 8/93 (8.6) | 8/31 (25.8) |  | 6/93 (6.5) | 8/31 (25.8) |  | 2/47 (4.3) | 1/10 (10.0) |
|  | Nuwara Eliya | 2/123 (1.6) | 31/127 (24.4) |  | 2/123 (1.6) | 29/127 (22.8) |  | 1/123 (0.8) | 7/127 (5.5) |
| **Total** |  | **29/351 (8.3)** | **96/294 (32.7)** |  | **9/351 (2.6)** | **65/294 (22.1)** |  | **3/305 (1.0)** | **14/273 (5.1)** |
| West | Gampaha | 1/3 (33.3) | 64/160 (40.0) |  | 1/3 (33.3) | 54/160 (33.8) |  | 1/3 (33.3) | 5/160 (3.2) |
|  | Colombo | 25/150 (16.7) | 35/101 (34.7) |  | 18/141 (12.8) | 17/83 (20.5) |  | 0/141 (0) | 6/83 (7.3) |
|  | Kalutara | 0/97 (0) | 17/117 (14.0) |  | 0/88 (0) | 6/117 (5.1) |  | 0/88 (0) | 1/116 (0.9) |
| **Total** |  | **26/250 (10.4)** | **116/378 (30.7)** |  | **19/232 (8.2)** | **77/360 (21.4)** |  | **1/232 (0.4)** | **12/360 (3.4)** |
| Sabaragamuwa | Ratnapura | 0/90 (0) | 10/139 (7.2) |  | 0/90 (0) | 6/139 (4.3) |  | 0/90 (0) | 1/139 (0.7) |
|  | Kegalle | 1/95 (1.1) | 50/138 (36.2) |  | 1/95 (1.1) | 31/137 (22.6) |  | 1/85 (1.2) | 10/136 (7.4) |
| **Total** |  | **1/185 (0.5)** | **60/277 (21.7)** |  | **1/185 (0.5)** | **37/276 (13.4)** |  | **1/175 (0.6)** | **11/275 (4.0)** |
| Uva | Badulla | 30/180 (16.7) | 31/118 (26.5) |  | 10/180 (5.6) | 20/117 (17.1) |  | 0/180 (0) | 2/117 (1.7) |
|  | Moneragala | 13/118 (11.0) | 10/66 (15.2) |  | 7/118 (5.9) | 8/66 (12.1) |  | 0/118 (0) | 1/66 (1.5) |
| **Total** |  | **43/298 (14.4)** | **41/184 (22.3)** |  | **17/298 (5.7)** | **28/183 (14.6)** |  | **0/298 (0)** | **3/183 (1.7)** |
| South | Galle | 1/59 (1.7) | 25/67 (37.3) |  | 0/59 (0) | 23/67 (34.3) |  | 0/59 (0) | 2/67 (3.0) |
|  | Hambantota | 18/130 (13.8) | 55/122 (45.1) |  | 9/130 (6.9) | 36/122(29.5) |  | 0/130 (0) | 3/122 (2.5) |
|  | Matara | 10/66 (15.2) | 23/101 (22.8) |  | 10/66 (15.2) | 14/92 (15.2) |  | 0/66 (0) | 2/92 (2.2) |
| **Total** |  | **29/255 (11.4)** | **103/290 (34.4)** |  | **19/255 (7.5)** | **73/281 (26.0)** |  | **0/255 (0)** | **7/281 (2.5)** |
| **Overall total** |  | **322/2877 (11.2)** | **783/2886 (27.1)** |  | **165/2854 (5.8)** | **492/2840 (17.3)** |  | **28/2785 (1.0)** | **130/2794 (4.6)** |
